# Cross-cultural measurement equivalence of the 5-level EQ-5D (EQ-5D-5L) in patients with type 2 diabetes mellitus in Singapore

**DOI:** 10.1186/s12955-015-0297-2

**Published:** 2015-07-16

**Authors:** Ye Wang, Ngiap-Chuan Tan, Ee-Guan Tay, Julian Thumboo, Nan Luo

**Affiliations:** Center for Surgery and Public Health, Brigham and Women’s Hospital, Harvard Medical School, 1620 Tremont Street, Boston, MA USA; SingHealth Polyclinics, 167 Jalan Bukit Merah, Singapore, Singapore; Singapore General Hospital, Outram Road, Singapore, Singapore; Yong Loo Lin School of Medicine, National University of Singapore, 1E Kent Ridge Road, Singapore, Singapore; Saw Swee Hock School of Public Health, National University of Singapore, 12 Science Drive 2, Block MD 1, 117597 Singapore, Singapore

**Keywords:** Cross-cultural measurement equivalence, Diabetes mellitus, EQ-5D-5L, Singapore

## Abstract

**Background:**

This study aimed to assess the measurement equivalence of the 5-level EQ-5D (EQ-5D-5L) among the English, Chinese, and Malay versions.

**Methods:**

A convenience sample of patients with type 2 diabetes mellitus were enrolled from a public primary health care institution in Singapore. The survey questionnaire comprised the EQ-5D-5L and questions assessing participants’ socio-demographic and clinical characteristics. Multiple linear regression models were used to assess the difference in EQ-5D-5L index (calculated using an interim algorithm) and EQ-visual analog scale (EQ-VAS) scores across survey language (Chinese vs. English, Malay vs. English, and Malay vs. Chinese). Measurement equivalence was examined by comparing the 90 % confidence interval of difference in the EQ-5D-5L index and EQ-VAS scores with a pre-determined equivalence margin. Multiple logistic regression models were used to assess the response patterns of the 5 Likert-type items of the EQ-5D-5L across survey language.

**Results:**

Equivalence was demonstrated between the Chinese and English versions and between the Malay and English versions of the EQ-5D-5L index scores. Equivalence was also demonstrated between the Chinese and English versions and between the Malay and Chinese versions of the EQ-VAS scores. Equivalence could not be determined between the Malay and Chinese versions of the EQ-5D-5L index score and between the Malay and English versions of the EQ-VAS score. No significant difference was found in responses to EQ-5D-5L items between any languages, except that patients who chose to complete the Chinese version were more likely to report “no problems” in mobility compared to those who completed the Malay version of the questionnaire.

**Conclusions:**

This study provided evidence for the measurement equivalence of the different language versions of EQ-5D-5L in Singapore.

## Introduction

In outcomes research, measurement equivalence is achieved if a scale generates comparable scores for individuals at the same level of health regardless of the populations they come from [1]. It is important to test the cross-cultural measurement equivalence of a self-reported health-status scale that is intended to compare health outcomes of populations from different cultures. This is because individuals from different cultures may have different ways of living, thinking, and expressing [2], leading to culture-specific interpretation of questionnaire items and/or response styles and difference in scale scores. When such difference is large enough, measurement equivalence cannot be assumed.

The 5-level EQ-5D (EQ-5D-5L) is a new version of the EQ-5D, a brief, generic health-status instrument [3]. It has been shown to have good psychometric properties [4–6] and suffer from fewer ceiling effects than the original version, i.e., the 3-level EQ-5D (EQ-5D-3L) [4–7]. The first part of the instrument contains 5 five-point Likert-type items (no/slight/moderate/severe/extreme), which describe five dimensions of a respondent’s health status on the day of the survey, i.e., mobility, self-care, usual activities, pain/discomfort, and anxiety/depression. An individual’s responses to the five items jointly form a multi-attribute health state for which a utility value (i.e., the EQ-5D-5L index score) can be assigned to indicate the utility of the health state to the general public [8]. The index score is anchored by 0 (death) and 1 (full health), with a higher score indicating higher utility. The second part is the EQ-visual analog scale (EQ-VAS), which is a vertical, 0 (the worst health state) to 100 (the best health state) hash-marked numerical rating scale, to rate respondents’ overall health.

The EQ-5D-5L questionnaire has been available in the official languages of Singapore, a multicultural, multiethnic city-state in South East Asia. Measurement equivalence is important to the use of health-related quality of life (HRQoL) instruments in Singapore because none of the official languages is spoken fluently by all residents, although many of them are multilingual. However, measurement equivalence of the EQ-5D-5L across different language subgroups of the Singaporean population is unknown. Therefore, this study aimed to assess the measurement equivalence of the EQ-5D-5L index score (calculated using an interim algorithm) and the EQ-VAS score among English, Chinese, and Malay versions in patients with type 2 diabetes mellitus (T2DM).

## Methods

### Patient recruitment

A cross-sectional survey was conducted in a convenience sample of T2DM patients visiting a primary health care institution in Singapore between July and December, 2012. Patients were enrolled if they were: 1) 21 years or older, 2) a Singaporean citizen or permanent resident, 3) diagnosed with T2DM, 4) able to read local newspapers or magazines in English, Chinese or Malay, and 5) able to see well enough to read text in the font size of 14.

### Data collection

Patients were approached by interviewers in the clinics while they were waiting for their routine consultations. Consenting patients were asked to complete the EQ-5D-5L questionnaire in English, Chinese or Malay, depending on their language preference. Patients’ socio-demographic and clinical characteristics were collected using a standardized questionnaire. The hemoglobin A1c (HbA1c) values of the patients were obtained from their doctors if they had the routine HbA1c test on the day of the survey. The HbA1c test measured the average blood glucose over the previous weeks and could give an indication of the long-term blood glucose control. This study was approved by the SingHealth Institutional Review Board.

### EQ-5D-5L index score

The index score was calculated using an algorithm, which can map each EQ-5D-5L health state to a linear combination of EQ-5D-3L health states [9] and thus the EQ-5D-3L value set [10], since values for EQ-5D-5L health states directly elicited from a representative general population sample were not available. The 5 Likert-type items of the EQ-5D-3L are similar to those of the EQ-5D-5L except that they only have three descriptive levels (no/moderate/extreme). We used the UK value set [11] due to the lack of a Singaporean value set at the time of this study.

### Statistical analysis

Descriptive statistics were used to describe participants’ characteristics and responses to the Likert-type items, the EQ-5D-5L index score, and the EQ-VAS score. Responses to the Likert-type items of the EQ-5D-5L were coded into “no(t)” = 0, “slight(ly)” = 1, “moderate(ly)” = 2, “severe(ly)” = 3 and “unable”/”extreme(ly)” = 4, and were compared across language using the Kruskal-Wallis test. The Chi-square test was used for other categorical variables, nominal or ordinal, and the ANOVA test for at least interval variables.

Three multiple linear regression models were used to estimate the between-language (Chinese vs. English, Malay vs. English, and Malay vs. Chinese) difference in the EQ-5D-5L index score, adjusting for age, gender, marital status (married vs. single vs. divorced/separated/widowed), employment status (employed/retired vs. unemployed/homemaker/others), housing type (government-subsidized house with 1–3 rooms vs. government-subsidized house with ≥ 4 rooms/private house), education (≤ secondary vs. > secondary school), HbA1c, body mass index, duration of T2DM (< 5 vs. ≥ 5 years), presence of T2DM-related complications (no vs. yes), and presence of comorbidities (< 2 vs. ≥ 2). Survey language was coded into dummy variables. This multiple linear regression analysis was also performed for the EQ-VAS score.

**Fig. 1:**
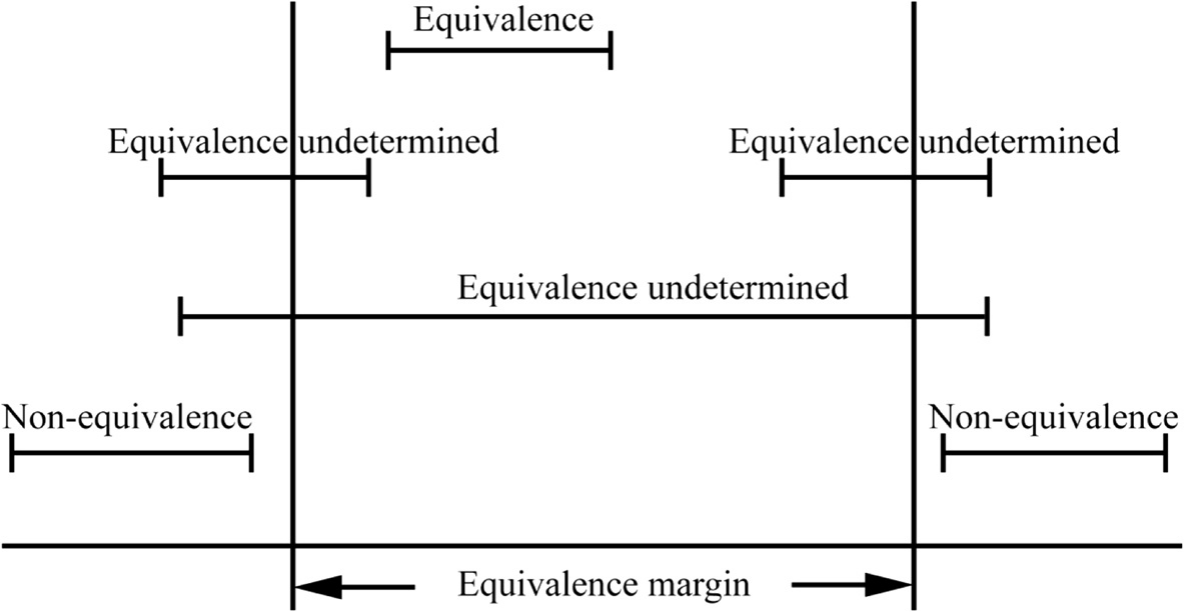
Possible relationships between equivalence margins and 90 % confidence intervals

Measurement equivalence is demonstrated if the difference across language is clinically unimportant [12]. Based on the approach to evaluating therapeutic equivalence in clinical trials [13, 14], we assessed measurement equivalence across language by comparing the 90 % confidence interval (CI) of the between-language difference in the EQ-5D-5L index and EQ-VAS scores, respectively, with a pre-determined equivalence margin that represented a range of score difference too small to be clinically important [15]. Based on studies of the minimally important differences of the EQ-5D [16–18], the equivalence margin was set as −0.08 to 0.08 for the EQ-5D-5L index scores [19, 20] and −10.00 to 10.00 for the EQ-VAS scores. This would lead to one of the three possible results (Fig. 1): 1) *‘equivalence’* was demonstrated if the 90 % CI fell completely within the equivalence margin, 2) ‘*equivalence undetermined’* (i.e., equivalence cannot be determined, and either equivalence or non-equivalence might be presented) was demonstrated if the 90 % CI partially overlapped with the equivalence margin, and 3)’*non-equivalence’* was demonstrated if the 90 % CI fell completely outside the equivalence margin.

In addition, fifteen multiple logistic regression models, five for each pair of survey languages, were used to compare the response patterns of participants to the EQ-5D-5L items between languages, with and without adjustment of the above-mentioned covariates. In all the models, the response of ‘no(t)’ was coded as 1 (the event) while the response of ‘slight(ly)’ , ‘moderate(ly)’, ‘severe(ly)’ or ‘unable’/’extreme(ly)’ was coded as 0 (the non-event). Survey language was coded into dummy variables.

Statistical tests were two-sided and performed using STATA/SE 11 software (StataCorp, Texas 77845 USA, 1984–2009), with the level of significance set at p < 0.05.

## Results

Seven hundred and twenty-nine patients participated in the study, representing an overall response rate of 61.5 %. Participants completing different language versions differed in some characteristics (Table 1). More severe levels of problems were less endorsed by all language groups for all health dimensions of the EQ-5D-5L (Table 2). Participants completing the Malay version (mean ± standard deviation [SD]: 81.85 ± 15.04) had significantly higher mean EQ-VAS score than those who completed the English (mean ± SD: 75.46 ± 18.46) or Chinese (mean ± SD: 78.00 ± 18.33) version (p < 0.001). No statistically significant difference was found in the trend of responses and the mean EQ-5D-5L index score across language.

**Table 1:** Characteristics of participants

	Survey language			
Characteristic, n (%)^a^	English (*n* = 311)	Chinese (*n* = 200)	Malay (*n* = 218)	p value^b^
Age (years), mean (SD)	56.31 (11.46)	59.98 (10.06)	54.62 (10.36)	< 0.001
Gender				< 0.001
Male	168 (54.0)	90 (45.2)	78 (35.8)	
Female	143 (46.0)	109 (54.8)	140 (64.2)	
Ethnicity				< 0.001
Chinese	185 (59.5)	200 (100.0)	2 (0.9)	
Malay	53 (17.0)	0 (0.0)	209 (95.9)	
Indian	73 (23.5)	0 (0.0)	7 (3.2)	
Marital status				0.020
Married	225 (72.4)	158 (79.0)	182(83.9)	
Divorced/separated/widowed	39 (12.5)	24 (12.0)	17 (7.8)	
Single	47 (15.1)	18 (9.0)	18 (8.3)	
Employment status				< 0.001
Employed	157 (50.5)	72 (36.2)	101 (46.5)	
Retired	77 (24.8)	66 (33.2)	10 (4.1)	
Unemployed	11 (3.5)	4 (2.0)	11 (5.1)	
Homemaker	52 (16.7)	49 (24.6)	88 (40.6)	
Others	14 (4.5)	8 (4.0)	7 (3.2)	
Housing type				0.141
GSH with 1–3 rooms	102 (32.9)	76 (38.0)	63 (28.9)	
GSH with ≥ 4 rooms/private house	208 (67.1)	124 (62.0)	155 (71.1)	
Education				< 0.001
No formal qualification	44 (14.2)	37 (18.7)	83 (38.1)	
Primary school	46 (14.8)	67 (33.8)	49 (22.5)	
Secondary school	125 (40.2)	67 (33.8)	53 (24.3)	
Post-secondary	96 (30.9)	27 (13.6)	33 (15.1)	
HbA1c (%), mean (SD)	7.59 (1.72)	7.34 (1.15)	6.78 (2.06)	< 0.001
BMI (kg/m^2^) , mean (SD)	27.21 (6.24)	25.19 (4.34)	27.32 (6.08)	< 0.001
Duration of having T2DM (years)				0.663
< 5	127 (41.1)	84 (42.2)	83 (38.1)	
≥ 5	182 (58.9)	115 (57.8)	135 (61.9)	
Presence of T2DM-related complications^c^				< 0.001
No	223 (71.7)	111 (55.5)	164 (75.2)	
Yes	88 (28.3)	89 (44.5)	54 (24.8)	
Presence of comorbidities^d^				< 0.001
< 2	156 (50.2)	65 (32.5)	86 (39.5)	
≥ 2	155 (49.8)	135 (67.5)	132 (60.6)	

*BMI* body mass index; *HbA1c* hemoglobin A1c; *SD* standard deviation; *GHS* government-subsidized house; *T2DM* type 2 diabetes mellitus

^a^Data for each variable are reported in terms of n (%) unless otherwise stated

^b^The Chi-square test was used for categorical variables and the ANOVA test for continuous variables

^c^Complications included stroke, ischemic heart disease, kidney disease, peripheral neuropathy, eye disease, and peripheral vascular disease

^d^Comorbidities included cancer, arthritis, hypertension, high blood cholesterol, asthma, bronchitis, liver disease, metal disorders, urology problems, ear/nose/throat problems, and other chronic conditions

**Table 2:** Distributions of EQ-5D-5L dimension, index and VAS scores by survey language

			Survey language			
Measure	Level of problems	Total (*n* = 729)	English (*n* = 311)	Chinese (*n* = 200)	Malay (*n* = 218)	p value^a^
Dimension score, n (%)						
Mobility	No	539 (73.9)	238 (76.5)	152 (76.0)	149 (68.4)	0.054
	Slight	104 (14.3)	43 (13.8)	29 (14.5)	32 (14.7)	
	Moderate	55 (7.5)	16 (5.1)	12 (6.0)	27 (12.4)	
	Severe	21 (2.9)	10 (3.2)	4 (2.0)	7 (3.2)	
	Unable	10 (1.4)	4 (1.3)	3 (1.5)	3 (1.4)	
Self-care	No	624 (85.6)	272 (87.5)	169 (84.5)	183 (83.9)	0.363
	Slight	58 (8.0)	27 (8.7)	16 (8.0)	15 (6.9)	
	Moderate	30 (4.1)	8 (2.6)	10 (5.0)	12 (5.5)	
	Severe	15 (2.1)	4 (1.3)	4 (2.0)	7 (3.2)	
	Unable	2 (0.3)	0 (0.0)	1 (0.5)	1 (0.5)	
Usual activities	No	570 (78.2)	248 (79.7)	155 (77.5)	167 (76.6)	0.486
	Slight	97 (13.3)	47 (15.1)	22 (11.0)	28 (12.8)	
	Moderate	45 (6.2)	11 (3.5)	18 (9.0)	16 (7.3)	
	Severe	11 (1.5)	2 (0.6)	3 (1.5)	6 (2.8)	
	Unable	6 (0.8)	3 (1.0)	2 (1.0)	1 (0.5)	
Pain/discomfort	No	415 (57.0)	184 (59.2)	104 (52.3)	127 (58.3)	0.270
	Slight	222 (30.5)	89 (28.6)	67 (33.7)	66 (30.1)	
	Moderate	70 (9.6)	31 (10.0)	19 (9.6)	20 (9.2)	
	Severe	16 (2.2)	6 (1.9)	6 (3.0)	4 (1.8)	
	Extreme	5 (0.7)	1 (0.3)	3 (1.5)	1 (0.5)	
Anxiety/depression	Not	497 (68.4)	221 (71.1)	128 (64.7)	148 (67.9)	0.216
	Slightly	175 (24.1)	75 (24.1)	52 (26.3)	48 (22.0)	
	Moderately	42 (5.8)	11 (3.5)	15 (7.6)	16 (7.3)	
	Severely	10 (1.4)	4 (1.3)	2 (1.0)	4 (1.8)	
	Extremely	3 (0.4)	0 (0.0)	1 (0.5)	2 (0.9)	
Index score, mean (SD)		0.84 (0.22)	0.85 (0.20)	0.82 (0.23)	0.83 (0.22)	0.264
EQ-VAS score, mean (SD)		78.07 (17.65)	75.46 (18.46)	78.00 (18.33)	81.85 (15.04)	< 0.001

*EQ-5D-5L* 5-level EQ-5D; *EQ-VAS* EQ-visual analog scale; *SD* standard deviation

^a^The Kruskal-Wallis test was used for dimension scores and the ANOVA test for index and VAS scores

Adjusted and unadjusted results from the linear regression analyses are shown in Table 3. After adjusting for the covariates, the mean EQ-5D-5L index score of the Chinese version was higher than that of the English version; the Malay version had a lower mean EQ-5D-5L index score than the English and Chinese versions. Comparisons of the 90 % CIs of the differences with the respective pre-determined equivalence margin suggested that, equivalence of the EQ-5D-5L index scores was demonstrated between the Chinese and English versions and between the Malay and English versions, whereas equivalence could not be determined between the Malay and Chinese versions. The adjusted mean EQ-VAS scores of the Chinese and Malay versions were higher than that of the English version. The Malay version had a higher adjusted mean EQ-VAS score than the Chinese version. The 90 % CIs of the differences suggested that, while equivalence of the EQ-VAS scores was demonstrated between the Chinese and English versions and between the Malay and Chinese versions, equivalence could not be determined between the Malay and English versions.

**Table 3:** The 90 % confidence intervals of the differences in EQ-5D-5L index and VAS scores between different language groups

Measure	Unadjusted difference (90 % CI)^a^			Adjusted difference (90 % CI)^a^		
	Chinese vs. English^b^	Malay vs. English^b^	Malay vs. Chinese^c^	Chinese vs. English^b^	Malay vs. English^b^	Malay vs. Chinese^c^
EQ-5D-5L index score	−0.029 (−0.061 to 0.004)	−0.024 (−0.055 to 0.007)	0.004 (−0.033 to 0.042)	0.014 (−0.029 to 0.058)^d^	−0.009 (−0.050 to 0.031)^d^	−0.044 (−0.094 to 0.006)^e^
EQ-VAS score	2.549 (−0.064 to 5.163)	6.393 (3.850 to 8.936)	3.844 (1.146 to 6.541)	6.176 (2.615 to 9.738)^d^	7.572 (4.205 to 10.939)^e^	0.603 (−2.949 to 4.155)^d^

*CI* confidence interval; *EQ-5D-5L* 5-level EQ-5D; EQ-VAS, EQ-visual analog scale

^a^Linear regression was used; difference = the 1st group – the 2nd group (reference group)

^b^English version was the reference group

^c^Chinese version was the reference group

^d^Equivalence was demonstrated

^e^Equivalence cannot be determined

Adjusted and unadjusted results from the logistic regression analyses are presented in Table 4. After adjusting for the covariates, participants completing the Malay version were less likely to report ‘no problems’ in mobility than those completing the Chinese version (adjusted odds ratio: 0.435; 95 % CI: 0.221 to 0.855). Other between-language differences were not statistically significant.

**Table 4:** Odds ratios of reporting problems in EQ-5D-5L dimensions between different language groups

Dimension	Unadjusted OR (95 % CI)^a^			Adjusted OR (95 % CI)^a^		
	Chinese vs. English^b^	Malay vs. English^b^	Malay vs. Chinese^c^	Chinese vs. English^b^	Malay vs. English^b^	Malay vs. Chinese^c^
Mobility	0.971 (0.640 to 1.474)	0.662 (0.450 to 0.976)	0.682 (0.443 to 1.051)	1.589 (0.847 to 2.982)	0.882 (0.500 to 1.557)	0.435 (0.221 to 0.855)
Self-care	0.782 (0.470 to 1.301)	0.750 (0.458 to 1.228)	0.959 (0.566 to 1.624)	1.741 (0.808 to 3.751)	1.211 (0.604 to 2.429)	0.634 (0.282 to 1.427)
Usual activities	0.875 (0.568 to 1.348)	0.832 (0.548 to 1.264)	0.950 (0.602 to 1.501)	1.600 (0.836 to 3.060)	1.105 (0.615 to 1.986)	0.566 (0.284 to 1.128)
Pain/discomfort	0.756 (0.528 to 1.081)	0.963 (0.717 to 1.294)	1.275 (0.866 to 1.877)	0.826 (0.478 to 1.428)	1.157 (0.681 to 1.967)	1.107 (0.609 to 2.012)
Anxiety/depression	0.745 (0.509 to 1.090)	0.861 (0.592 to 1.253)	1.156 (0.770 to 1.737)	0.741 (0.415 to 1.321)	1.115 (0.629 to 1.975)	1.498 (0.789 to 2.843)

*CI* confidence interval; *EQ-5D-5L* 5-level EQ-5D; *OR* odds ratio

^a^Logistic regression was used; the event was endorsing the response option of “no(t)” as opposed to “slight(ly)”, “moderate(ly)”, “severe(ly)”, or “unable”/ “extreme(ly)”

^b^English version was the reference group

^c^Chinese version was the reference group

## Discussion

Assessment of self-reported health outcomes in Singapore usually involves multiple ethnic groups, which necessitates the use of more than one survey language. Therefore, only cross-culturally equivalent instruments would provide the most valid measurement in such a setting. In this study, measurement equivalence was found between the Chinese and English versions and between the Malay and English versions of the EQ-5D-5L index scores. Measurement equivalence was also found between the English and Chinese versions and between the Malay and Chinese versions of the EQ-VAS scores. The findings are consistent with a previous study, which reported that the 90 % CI of difference in EQ-5D-5L index and EQ-VAS scores between the Chinese and English versions were −0.02 to 0.06 and −5.30 to 5.50, respectively [21]. However, it should be noted that the Chinese and English EQ-5D-5L questionnaires used in that study were not official versions, although the study participants were Singaporean residents.

Nevertheless, equivalence of the EQ-5D-5L index scores could not be determined between the Malay and Chinese versions. Participants using the Chinese version reported better overall health status. This is consistent with previous studies, which found that ethnic Chinese were more likely to endure health problems than other ethnicities [22, 23]. Indeed, our analyses of the participants’ response patterns to the EQ-5D-5L items suggested that participants using the Chinese version, who were all ethnic Chinese, were less likely to report mobility problems than those using the Malay version, who were mainly ethnic Malay and Indian. Equivalence of the EQ-VAS scores between the Malay and English versions could not be confirmed. Patients completing the Malay version had higher adjusted mean EQ-VAS score than those completing the English version, indicating that the former would give higher rates to their overall health than the latter even if they were in same level of health. One explanation could be that the EQ-VAS has been found to be a more mental than physical health measure [24]; studies conducted in Singapore consistently found that Malays reported better mental health than other ethnicities [23, 25].

It should be noted that a comprehensive assessment of the cross-cultural measurement equivalence of the EQ-5D-5L should also include responses to the five items. The individual EQ-5D-5L items have also been used as independent outcome measures, and their cross-cultural equivalence cannot be inferred from that of the index or VAS score. Assessing the equivalence of the items, however, would require a sample size larger than the one we used in the current study. Therefore, we did not perform the equivalence analysis for the EQ-5D-5L items; it would not be informative to conclude that the equivalence of the items between any two language versions cannot be determined. The cross-cultural equivalence of the EQ-5D-5L at the item level should be examined in the future when suitable datasets are available.

This study has a few limitations. First, the convenience sample used in this study may have led to selection bias, as patients who had poorer health may have been less willing to participate in the survey. Second, the EQ-5D-5L index score was calculated using an interim algorithm, mapped to the general UK population-based EQ-5D-3L value set, which may not fully reflect the measurement properties of the index score obtained from direct valuation of the EQ-5D-5L health states. Third, most clinical data (e.g., presence of T2DM-related complications and comorbidities) used in this study were patient-reported, which may not be accurate.

In conclusion, this study provides evidence for the measurement equivalence of the EQ-5D-5L instruments across language, in a multicultural, multiethnic Asian population with T2DM. Future studies are needed to investigate the cross-cultural measure equivalence of the EQ-5D-5L items and whether this research finding can be generalized to other populations.

## Abbreviations

CI: Confidence interval
EQ-5D-3L: 3-level EQ-5D
EQ-5D-5L: 5-level EQ-5D
EQ-VAS: EQ-visual analog scale
HRQoL: Health-related quality of life
T2DM: Type 2 diabetes mellitus
